# Evaluation of a Novel Three-Dimensional Emergency Cricothyrotomy Task Trainer

**DOI:** 10.7759/cureus.14775

**Published:** 2021-04-30

**Authors:** Tina T Jing, Sharan J Shah, Nicholas B Dadario, Farrukh N Jafri

**Affiliations:** 1 Department of Emergency Medicine, Albert Einstein College of Medicine, Bronx, USA; 2 Department of Otorhinolaryngology - Head and Neck Surgery, Montefiore Medical Center, Bronx, USA; 3 Department of Emergency Medicine, Robert Wood Johnson Medical School, New Brunswick, USA; 4 Department of Emergency Medicine, White Plains Hospital, White Plains, USA

**Keywords:** emergency, cricothyrotomy, airway, simulation, bleeding, education, training

## Abstract

Background

Emergency cricothyrotomy is a critical, yet infrequently performed and time-sensitive procedure that requires practice in order to be reliably completed in emergent airway situations. Many physicians never have the opportunity to practice this rarely performed but highly impactful procedure during their training due to a lack of an affordable, high-fidelity training model. In this study, the educational impact and realism of a new synthetic cricothyrotomy training model (high-fidelity emergency cricothyrotomy, HiFEC) were compared with those of a standard porcine explant model.

Methodology

A total of thirty-one attending physicians from four medical specialties were recruited on a volunteer basis to participate in a cricothyrotomy simulation workshop. Participants were randomly assigned to complete the initial workshop using one of the two models but had the opportunity to practice on both trainers. Pre- and post-workshop comfort level as well as the realism of the models were surveyed using questionnaires and evaluated using a five-point Likert scale.

Results

Improvements in self-reported comfort levels were seen in both the porcine group (p = 0.0014) and HiFEC group (p = 0.0036) as well as overall (p < 0.001). The realism rating of both training models was similar with a median score of 4 on a five-point Likert scale. When comparing the cost of conducting our workshop using these models, the synthetic model saved over $650.

Conclusions

Given the similar realism of the models and the improvement in participant comfort level, the synthetic HiFEC trainer is an effective and more affordable alternative training model for emergency cricothyrotomies.

## Introduction

Difficult airway management is a critical skill for physicians in operating rooms, emergency departments (ED), and intensive care units (ICU) [1,2]. Failure to accurately identify and manage an airway obstruction can lead to serious consequences including significant morbidity and mortality for the patient [3,4]. A prior study found that difficult intubation with difficult ventilation occurs in 1.5% of procedures in operating rooms, impossible intubation with difficult ventilation in 0.3% of procedures, and a “cannot ventilate, cannot intubate” situation in 0.07% of procedures [5]. In situations where a patient is unable to be oxygenated via mask ventilation, supraglottic airway device, or tracheal tube, the clinician must turn to surgical airways such as tracheostomy and cricothyrotomy [6,7]. Emergency cricothyrotomy is a time-sensitive procedure that is rarely performed, but its correct execution is of critical importance when needed. It requires practice and preparation in order to be reliably performed in emergent airway situations. Unfortunately, many physicians never have the opportunity to practice this procedure during their training. One survey found that 48% of graduating emergency medicine residents had witnessed a cricothyrotomy and only 22% of emergency medicine graduates had performed a cricothyrotomy on a living patient [8]. Given this, it is crucial to optimize practice and training methods in safely and correctly performing an emergent cricothyrotomy.

Due to the low incidence of complete airway obstruction requiring a cricothyrotomy, simulation models and task trainers are required to develop clinical expertise to be able to perform this life-saving procedure successfully in an emergency [9,10]. Animal explant models, such as porcine tracheas, are considered the current standard, but these task trainers pose a number of problems. They can be expensive to obtain and difficult to store as they are considered a biohazard. Additionally, animal models can degrade quickly as they are a biological tissue model; hence, organizers of cricothyrotomy trainings using a porcine model have a short window of time to run the workshops. Furthermore, there is a risk of the animal tissue being infected with a transmissible virus or bacteria that can then expose a participant in the case of a sharps injury during the training session. Other high-fidelity models, like human cadavers, are even more difficult to obtain and store. Synthetic options range from cheap, low-fidelity options to very expensive, high-fidelity trainers. However, these current models fail to capture a crucial aspect of the real procedure - the profuse bleeding that may prevent direct visualization of the procedure.

The aim of this study was to assess the educational experience and fidelity of a new synthetic, high-fidelity emergency cricothyrotomy (HiFEC) model in comparison to a standard porcine tissue explant training model.

## Materials and methods

This was a crossover study using a convenience sample of attending physicians. The study was deemed exempt as an educational intervention by the institutional review board at White Plains Hospital. Oral consent was obtained from all participants. This study follows the Strengthening the Reporting of Observational Studies in Epidemiology guidelines [11].

A total of 31 attending physicians were recruited on a voluntary basis via email between September 2019 and May 2020 to participate in a difficult airway simulation workshop. Email announcements were sent to various departments at White Plains Hospital, a community hospital affiliated with the Montefiore Health System in White Plains, NY, USA. All attending physicians who care for patients with airway management were invited to participate in the study regardless of medical specialty or clinical experience.

**Figure 1:**
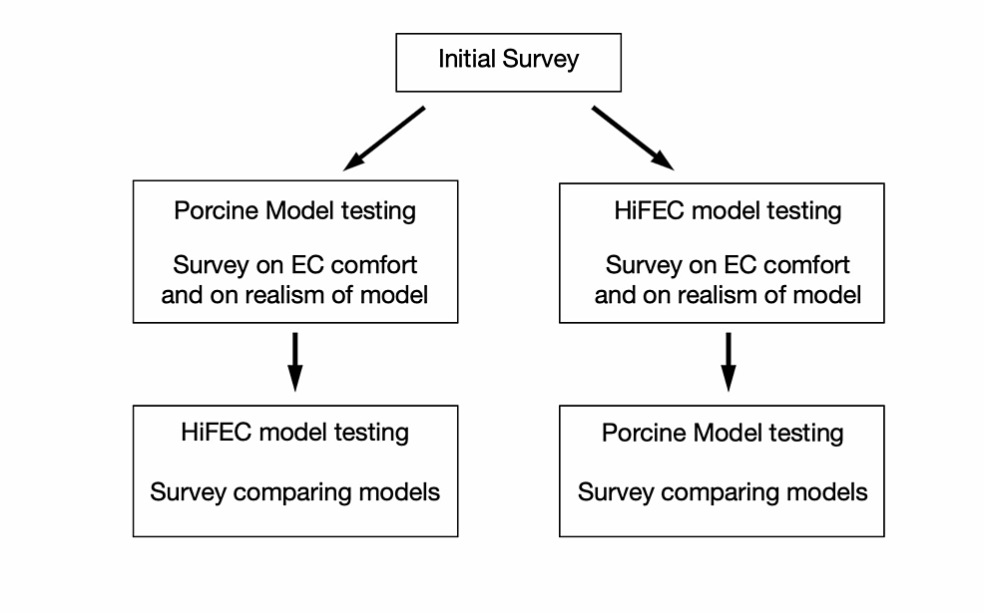
Flowchart of the study design. HiFEC: high-fidelity emergency cricothyrotomy; EC: emergency cricothyrotomy

Upon arrival, participants completed an initial survey evaluating demographic information, their prior experience with emergency cricothyrotomies, and their comfort level performing them. Survey questions were formatted using a conventional five-point Likert scale. An instructor reviewed the key steps to performing an emergency cricothyrotomy, including identifying anatomical landmarks, dilation of the incision, and tube insertion. Participants were then randomly assigned to perform an emergency cricothyrotomy using a porcine model or the HiFEC model (see below for model details). Following this, participants were asked to assess the model they had just used and were again queried regarding their comfort performing emergency cricothyrotomies. They then had the opportunity to practice emergency cricothyrotomies using both training models and were again asked to evaluate the models on a final survey (Figure 1).

The primary outcome measure was participants’ self-reported comfort level performing emergency cricothyrotomies after the emergency airway management scenario compared to their comfort level prior to the workshop. A secondary outcome measure was the realism rating of the training models used in the workshop.

Statistical analysis

Anonymized data were entered into a Microsoft Excel® (Microsoft Corporation, Redding, WA, USA) spreadsheet and analyzed. Data were summarized using median and range. As the data were non-parametric, a paired Wilcoxon signed-rank test with continuity correction with α = 0.05 was performed using RStudio (RStudio Team, Boston, MA, USA) to compare the difference in comfort level before and after the workshop for the entire group, HiFEC group, and porcine group [12].

The high-fidelity emergency cricothyrotomy model

**Figure 2:**
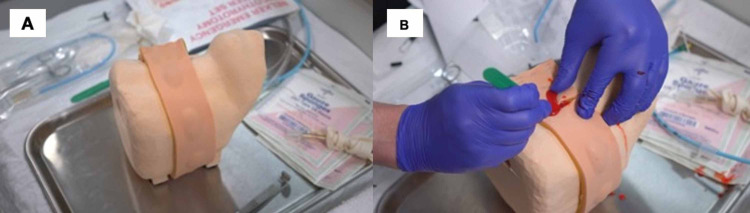
Images of the HiFEC model. (A) Before and (B) after incision for an emergency cricothyrotomy HiFEC: high-fidelity emergency cricothyrotomy

The HiFEC model consists of a piece of synthetic skin on top of a sealed, pressurized, enclosed plastic membrane. The exterior surface of this membrane is thinner and represents subdermal fascia and fat. Within the pressurized enclosure is a small volume of synthetic blood, which is released once the membrane is incised. This bleeding obscures the learner’s view of the anatomical structures beneath the incision, which better recapitulates the conditions during a real emergency cricothyrotomy. Below this lies the thicker, interior surface of the plastic membrane that mimics the cricothyroid membrane. Finally, there is adhesive backing on this portion of the model so that it can be easily removed and replaced for each learner. This also allows the skin and membrane to be pulled taut, making the incision more realistic. Beneath all this is a reusable model of the bony anatomy and landmarks, including the thyroid cartilage and cricoid cartilage (Figures 2A, 2B). The cost of the HiFEC trainer was $50 for the reusable base representing the bony anatomy and $33 for each adhesive strip that contains seven incision sites.

The porcine explant model

To create the porcine tracheostomy model, pig tracheas were purchased from a wholesale porcine manufacturer and shipped to the home of the workshop coordinator. The cost of the tracheas was $25 per trachea plus $16 for shipping and handling. For the 45 tracheas ordered on nine separate training days, the total cost was $1,269. They were shipped frozen and thus required thawing prior to use in the simulation. No other post-shipment processing was done to the tracheas. The porcine explant model includes the epiglottis, supraglottis, larynx, trachea down to carina, and proximal mainstem bronchi. The model includes just the visceral organs without the overlying skin or muscle. It does include the cricothyroid membrane, where the incision is made for cricothyrotomies, as well as the cricoid and thyroid cartilages, which are important anatomical landmarks needed to properly identify the cricothyroid membrane. Each porcine model can only be used once.

## Results

The 31 attending physicians recruited to this study came from four different medical specialties, had a wide range of work experience, and varied in prior experience performing emergency cricothyrotomies (Table 1).

**Table 1:** Demographic characteristics of participants by specialty. Values provided are either number (percentage) or median (range)

	Emergency Medicine	Anesthesiology	Intensive Care	Internal Medicine	Total
Participants	n = 15 (48.3%)	n = 14 (45.2%)	n = 1 (3.2%)	n = 1 (3.2%)	n = 31
Work experience (years)	9 (1.5–30)	11.5 (2–34)	2	7	9 (1.5–34)
Prior emergency cricothyrotomies	0 (0–4)	0	0	0	0 (0–4)

Following the simulated difficult airway scenario that involved performing an emergency cricothyrotomy using the randomized trainer, participants demonstrated an improvement in self-reported comfort level performing emergency cricothyrotomies in both the porcine (p = 0.0014) and HiFEC (p =0.0036) groups as well as overall (p < 0.001) compared to pre-workshop comfort levels (Table 2).

**Table 2:** Survey results on comfort level. Survey results comparing participants’ self-reported comfort level before the session and after the simulated difficult airway scenario using either the porcine or HiFEC model. Comparisons were done using a paired Wilcoxon signed-rank test. Comfort level was evaluated on a five-point Likert scale (1 = highly uncomfortable, 2 = uncomfortable, 3 = neutral, 4 = comfortable, 5 = highly comfortable) HiFEC: high-fidelity emergency cricothyrotomy

What is your level of comfort performing an emergency cricothyrotomy unassisted by another physician?							
	Survey response					Median	P-Value
	1	2	3	4	5		
Before (n = 31)	11	8	6	5	1	2	
After – Porcine (n = 16)	0	3	4	8	0	3.75	p = 0.0014
After – HiFEC (n = 15)	2	2	5	4	2	3	p = 0.0036
After – Total (n = 31)	2	5	9	12	2	3	p < 0.001

Participants rated the realism of the porcine and HiFEC models similarly with a median rating of 4 for both models (Table 3). When asked to compare the realism of the HiFEC model to that of the porcine model on a five-point Likert scale (1 = HiFEC highly inferior, 2 = HiFEC inferior, 3 = neutral, 4 = HiFEC superior, 5 = HiFE highly superior), participants rated the two trainers equally with a median rating of 3.

**Table 3:** Survey results on realism. Survey results of the realism rating for the porcine explant and the HiFEC training models. Realism was evaluated on a five-point Likert scale (1 = highly unrealistic, 2 = unrealistic, 3 = neutral, 4 = realistic, 5 = highly realistic) HiFEC: high-fidelity emergency cricothyrotomy

What is the realism of the training model?						
	Survey response					Median
	1	2	3	4	5	
Porcine (n = 16)	0	0	4	10	2	4
HiFEC ( n = 15)	0	1	4	7	3	4

Most participants (74.2%) indicated they preferred to train using the porcine model over the HiFEC model while 12.9% indicated they preferred the HiFEC model over the porcine model. Two participants (6.45%) preferred the porcine and HiFEC models equally, while one participant (3.22%) indicated they preferred a different three-dimensional model they had used previously.

## Discussion

Emergent cricothyrotomy is a low frequency but highly impactful procedure. Successful placement of a surgical airway at times when intubation and ventilation is not possible can lead to a lifesaving clinical outcome. However, due to its low frequency, cricothyrotomies must be practiced often by clinicians responsible for airway management using low-risk simulation models that are highly realistic and inexpensive in order to become more comfortable and competent when performing them in a clinical setting. There are a variety of cricothyrotomy simulators available for clinicians to practice on, including cadaver-based models, porcine models, live-animal models, and manufactured simulators. Cricothyrotomy training on porcine models has been documented and accepted as a training model for learning this infrequently performed procedure [13]. However, many of these models do not include a critical obstacle in doing a successful cricothyrotomy: intraoperative bleeding [14]. The HiFEC model has a compartment more superficial to the larynx and trachea which extrudes synthetic blood when incised. This recreates a crucial obstacle that clinicians may face while performing a cricothyrotomy.

After surveying 31 clinicians who are involved in airway management, this study found that participants were more comfortable performing a cricothyrotomy after practicing on a simulator regardless of simulator type, porcine or the HiFEC model. This is in line with other studies finding that simulation-based task training improves the comfort and confidence of participants for such low-frequency, high-impact procedures [15,16]. The authors will continue to run more cricothyrotomy task training simulation programs for clinicians in the hospital’s ED, as baseline comfort scores from this study were relatively low and simulation has proven to have a quantitative impact on comfort and confidence in approaching high stress situations.

Additionally, we also found that there was very little difference in the realism rating between the models. However, the majority of the subjects preferred the porcine model for simulations. Comments from the study participants indicated that they liked the realistic bleeding of the HiFEC model but found the tissue fidelity of the porcine model to be preferable when doing practice procedures. Many enjoyed the difficulty of using the porcine model, with the possibility to get into the wrong tissue plane and cut down on the wrong site or insert the endotracheal tube into a false passage, both real complications of emergent cricothyrotomies. This correlates with previous studies showing that porcine models were preferred over manikin models in terms of realistic simulation due to higher scores in feel of skin turgor and degree of difficulty of skin penetration [17].

Cost analysis and feasibility

The porcine model can only be utilized once per participant following a practice cricothyrotomy, while the HiFEC model contains seven practice sites. Therefore, to accurately compare the head-to-head cost of these two models, a 35-person program was analyzed despite only having 31 participants in this current study.

When only considering the costs of the model, the HiFEC model saves on average over $18.58 per person, or $650 per 35-person program. This number is increased because the porcine model also requires the purchase of cleaning supplies and equipment (i.e., scalpels) to utilize this training method, while these supplies are all included in a HiFEC training kit. Including the cost of these extra but necessary items, the savings from using the HiFEC model increases to on average $27.93 per person, or $977.55 per 35-person program. Furthermore, the porcine model requires (1) proper storage in a refrigerated cooler wherever possible, this was done by our instructor at the dispense of his home; and (2) proper biohazard disposal by outside services, the costs of which were not included in these analyses. In contrast, the HiFEC can be stored anywhere (i.e., a closet in this study) and disposed of by any methods as it is non-biohazard material. Thus, the HiFEC model is superior in terms of cost and likely more feasible for regular hospital training. This cost difference becomes even more important considering the repeated need for clinicians to practice this infrequently performed procedure in order to maintain their competence and comfort levels.

Another benefit to using the HiFEC model over the porcine model is to avoid potential exposure to biohazards in the porcine model. When working on a deceased animal model with a sharp instrument such as a scalpel, there is an inherent risk of exposing the participant to zoonotic biohazards that may be present in the tissue. The presence of zoonotic viruses and bacteria in pigs has been well documented [18], and the decrease of such exposure to participants is a valuable advancement in safely training clinicians.

Limitations and future directions

Limitations of our study include the relatively small sample size and participants recruited from only one hospital. Additionally, most of our participants had never performed an emergent bedside or intraoperative cricothyrotomy, making it difficult to compare any model in terms of realism of the simulation.

**Figure 3:**
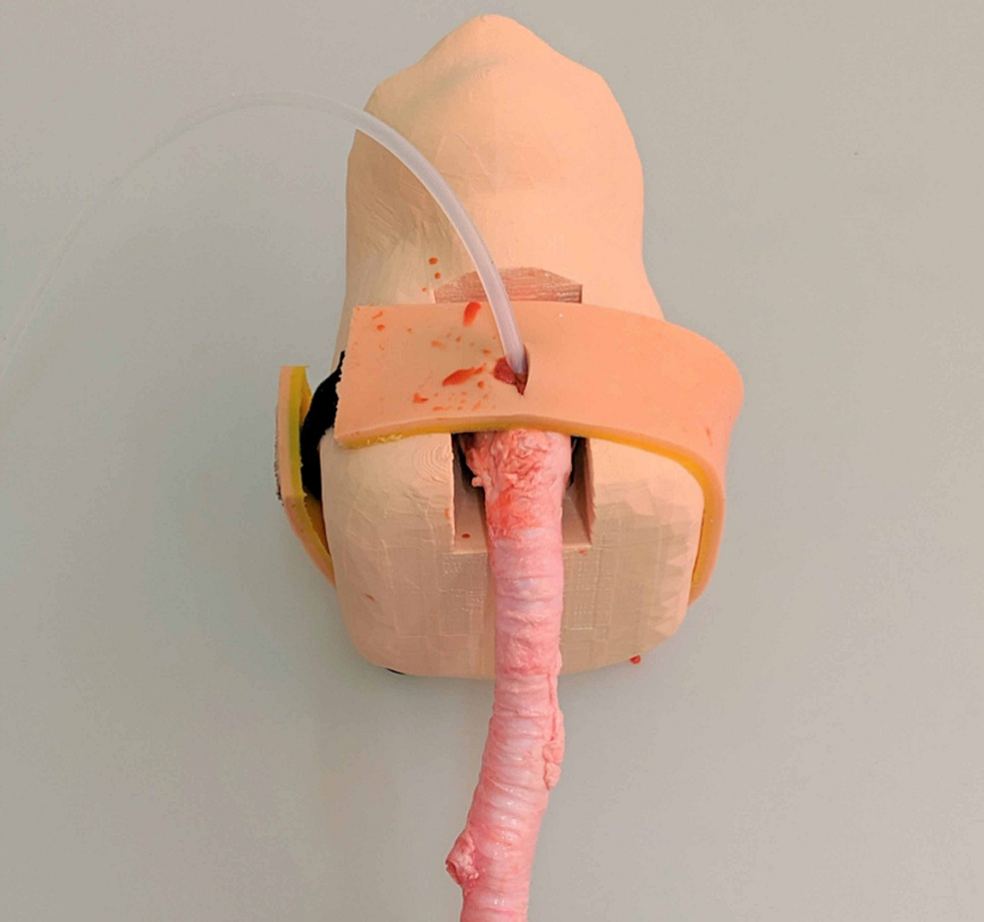
Image of the HiFEC model combined with a porcine explant trachea after an emergency cricothyrotomy. HiFEC: high-fidelity emergency cricothyrotomy

Future directions will be to design a model that recapitulates the tactile feel of real tissue with the possibility of creating false passages while also simulating bleeding. One option is to incorporate a porcine explant trachea with a synthetic three-dimensional model, which is currently under development (Figure 3). However, this combined model would have the same storage and biohazard concerns as the porcine explant model while being more expensive than the porcine model alone. Development of an emergency cricothyrotomy model that mimics live tissue, simulates bleeding, but avoids the high cost and storage of porcine models will be critical toward the advancement of realistic task trainers.

## Conclusions

This HiFEC trainer was rated similarly in realism to the current standard porcine explant model, and participants using this model demonstrated similar improvements in self-reported comfort level. Given the low incidence of this critical and time-sensitive procedure, it is important for clinicians to routinely practice emergency cricothyrotomies on a realistic but inexpensive model in order to be more comfortable and competent when performing them in real clinical settings. With its low cost and ease of storage, this synthetic, bleeding model is an effective alternative trainer for practicing emergency cricothyrotomy. Future models should aim to capture the tactile feel of live tissue while integrating simulated bleeding to provide realistic experiences for participants while avoiding the cost and storage complications associated with animal products.
